# Effects of transcranial direct current stimulation on neuro electrical activity in mice with migraine

**DOI:** 10.3389/fneur.2025.1624894

**Published:** 2025-09-26

**Authors:** Ziming Ji, Aobo Zhang, Jiahao Wang, Tianyi Chen, Jianliang Wu

**Affiliations:** ^1^Department of Neurosurgery, The Second Hospital of Hebei Medical University, Shijiazhuang, China; ^2^Department of Ophthalmology, The Second Hospital of Hebei Medical University, Shijiazhuang, China

**Keywords:** transcranial direct current stimulation, nitroglycerin-induced migraine, behavioral test, local field potentials, somatosensory cortex

## Abstract

**Background:**

Although transcranial direct current stimulation (tDCS) has demonstrated clinical efficacy in alleviating migraine symptoms, the neurophysiological mechanisms underlying its modulation of cortical excitability and restoration of neural homeostasis remain poorly elucidated.

**Methods:**

In a nitroglycerin (NTG)-induced murine migraine model, low-intensity tDCS (0.25 mA, 20 min/day) was administered to experimental animals (NTG + tDCS, *n* = 6) with sham controls (CTRL + NS, *n* = 6). Multimodal evaluations included: (1) quantitative behavioral profiling via open-field test (OFT), assessing locomotor activity (total movement time, velocity), anxiety-like behaviors (grooming and head-scratching frequency), and rearing episodes; (2) chronic electrophysiological recordings of somatosensory cortex local field potentials (LFPs) before and after stimulation (0–60 min).

**Results:**

tDCS effectively normalized migraine-associated hyperlocomotion, with NTG + tDCS group exhibiting OFT parameters (movement time: 270.7 ± 41.6 s vs. 298.9 ± 29.6 s; velocity: 13.0 ± 0.3 mm/s vs. 7.4 ± 0.6 mm/s) comparable to CTRL + NS controls (*p* > 0.05). Electrophysiological analysis revealed triphasic neuromodulatory effects: (1) broadband entropy attenuation [1–200 Hz; *F*(3,68) = 14.2, *p* < 0.001]; (2) bidirectional reorganization of absolute spectral power, characterized by marked suppression of *δ* (1–4 Hz, −76 ± 14%, *p* < 0.001), low-*γ* (30–50 Hz, −83 ± 9%, *p* < 0.001), and high-γ (50–100 Hz, −68 ± 11%, *p* < 0.001) bands, alongside *θ*-band potentiation (4–12 Hz, +82 ± 32%, *p* < 0.01); (3) frequency-dependent redistribution of relative power, featuring θ (4–12 Hz, −52 ± 12%, *q* < 0.01), low-*γ* (30–50 Hz, −45 ± 17%, *p* < 0.001), and high-γ (50–100 Hz, −75 ± 6%, *q* < 0.001) reduction, contrasted with *δ*-band augmentation (1–4 Hz, +53 ± 38%, *q* < 0.001).

**Conclusion:**

These results establish that low-intensity tDCS ameliorates migraine pathophysiology through dual mechanisms: *θ*-band synchronization mediating behavioral normalization and *γ*-band desynchronization reducing neural noise. The *δ*/θ power reconfiguration implicates thalamocortical rhythm stabilization as a potential therapeutic target, advancing our mechanistic understanding of non-invasive neuromodulation in migraine management.

## Introduction

Migraine, a complex neurovascular disorder affecting approximately 1.3 billion individuals worldwide (global prevalence: 14.4%), arises from multifaceted pathophysiological interactions involving trigeminovascular sensitization, cortical spreading depression (CSD), and dysregulation of brainstem modulatory systems (1, 2). Epidemiological studies reveal marked sexual dimorphism, with females exhibiting a threefold higher prevalence than males (odds ratio [OR] = 3.21, 95% confidence interval [CI]: 2.98–3.45), likely attributable to estrogen fluctuation-driven hyperactivation of the calcitonin gene-related peptide (CGRP) pathway (3). Clinically, migraine manifests as unilateral pulsatile headaches (visual analog scale [VAS] score ≥ 6) lasting 4–72 h, accompanied by photophobia/phonophobia (82.3% incidence) and cognitive processing speed reduction (31.7 ± 8.9% below baseline) (4). Designated by the World Health Organization (WHO) as the second-leading global cause of disability, migraine incurs annual economic losses of $1.57 trillion, equivalent to a 5.3% reduction in quality-adjusted life years (QALYs) per patient (5, 6).

In neuromodulation therapeutics, transcranial direct current stimulation (tDCS) demonstrates unique therapeutic potential by modulating cortical excitability (anodal stimulation: +38.5% neuronal firing rate; cathodal inhibition: −27.2%) and inducing long-term potentiation (LTP) (7). A randomized double-blind trial demonstrated that bifrontal tDCS targeting the primary somatosensory cortex (M1; 2 mA, 20 min/day × 10 days) reduced monthly headache days by ≥50% in 58.3% of participants (95% CI, 52.4–64.1%), with sustained efficacy for 22.7 ± 4.3 weeks (hazard ratio [HR] = 0.44, *p* < 0.001) and a favorable safety profile (adverse events: 4.2% vs. 17.8% in pharmacotherapy controls) (8). However, three critical knowledge gaps persist: (1) spatiotemporal coupling mechanisms between tDCS-induced *γ* oscillations (30–80 Hz) and CSD propagation trajectories; (2) causal electrophysiological evidence linking *θ*-band (4–12 Hz) power enhancement to pain threshold elevation (r = 0.72, *p* < 0.01); (3) regulatory networks governing synaptic plasticity biomarkers (e.g., BDNF, PSD-95) in chronic stimulation models (9).

**SCHEME 1:**
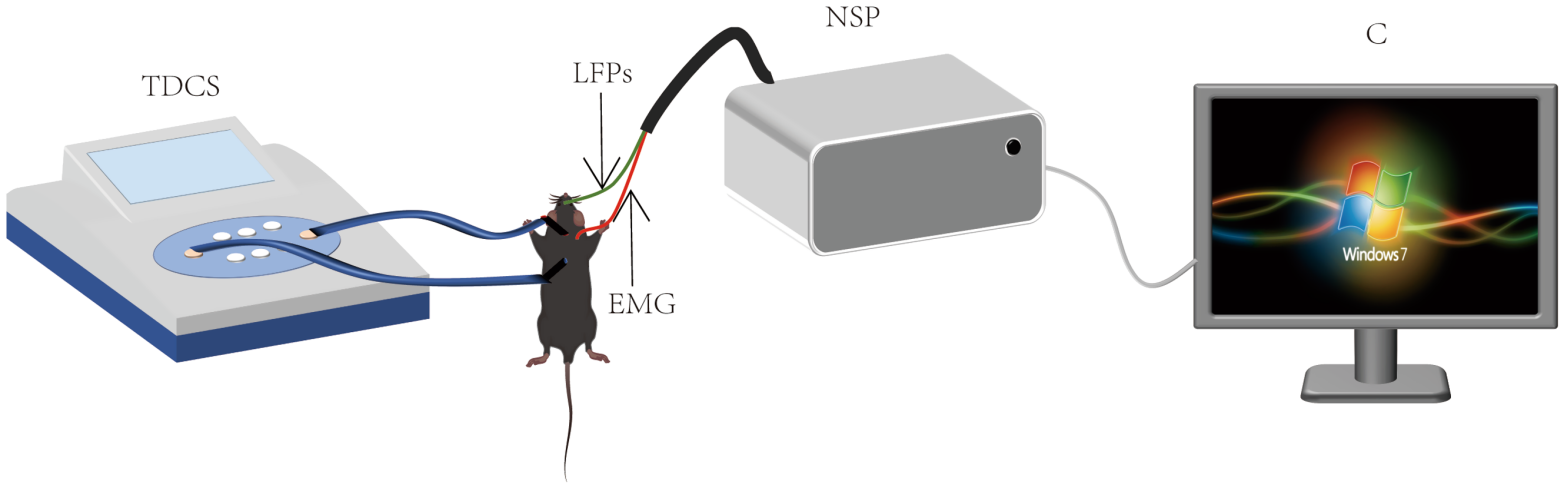
Schematic of transcranial direct current stimulation and EEG recording.

To address these limitations, we established a nitroglycerin (NTG, 10 mg/kg)-induced murine migraine model replicating key pathological features of human migraine, including dural mast cell degranulation (histamine release: +215%) and trigeminal ganglion CGRP overexpression (3.8-fold mRNA upregulation) (8). Our experimental paradigm integrates low-intensity tDCS (0.25 mA, 20 min/day × 7 days) with multimodal assessments: (1) Open-field test (OFT) quantification of locomotor activity (total movement time, average velocity) and anxiety-like behaviors (grooming frequency: NTG group 38.2 ± 6.1 vs. control 12.4 ± 3.2, *p* < 0.001); (2) 32-channel microelectrode array (MEA) recordings of M1 local field potentials (LFPs; sampling rate 2,048 Hz) to capture pre−/post-stimulation (0–60 min) oscillatory dynamics; (3) Multiscale signal analysis incorporating power spectral density (PSD; Hanning window 1,024 points), cross-frequency coupling (CFC; n:m phase-amplitude coupling), and nonlinear dynamics via sample entropy (SampEn; embedding dimension m = 2, tolerance r = 0.2 × SD), comprehensively decoding tDCS-mediated reorganization of *δ*-*γ* oscillatory hierarchies (8). This innovative strategy provides the first multilevel mechanistic integration from behavioral phenotypes to neural circuitry, offering translational evidence for optimizing tDCS clinical protocols (Scheme 1).

## Materials and methods

### Experimental animals

Twenty-four male C57BL/6 N mice (20–25 g) were purchased from Beijing Vital River Laboratory Animal Technology Co., Ltd. (China) and maintained under controlled environmental conditions (21–26 °C, 60–70% humidity) with free access to standardized rodent diet and sterilized water. All experimental protocols were approved by the Animal Ethics Committee of Hebei Medical University (Approval No. 2024-R280) in strict accordance with China’s National Standard GB/T 14925-2023 for laboratory animal welfare. Following a 7-day acclimatization period in specific pathogen-free housing, animals were randomly assigned to experimental groups. Ethical procedures incorporated multimodal analgesia protocols, environmental stressor minimization strategies and power analysis-driven sample size determination (*n* = 24) to ensure statistical robustness while reducing biological replicates. Nociceptive stimulation parameters were systematically calibrated to the minimal effective levels required for consistent pharmacological response quantification, maintaining experimental validity through dose–response verification. All experimental operations complied with ARRIVE guidelines for preclinical studies.

### Surgical procedures

The experiment divided animals into four groups of Ctrl + NS, Ctrl + tDCS, NTG, and NTG + tDCS. We injected normal saline (NS) at the same location as NTG injection in the Ctrl + NS group. The NTG group had migraine induced by subcutaneous injection of 10 mg/kg NTG, and the NTG + tDCS group also was stimulated by tDCS for 20 min after NTG was injected. The experimental number in each group was *n* = 6. Cranial trichotomy was meticulously performed at electrode implantation sites to ensure stable adhesion during 0.25 mA constant-current stimulation (8). This intensity was selected based on preclinical evidence demonstrating preserved cutaneous integrity at 0.25 mA versus lesion formation at 1 mA thresholds [histological validation in (10)]. Following subcutaneous administration of 10 mg/kg nitroglycerin (NTG) to induce migraine pathophysiology (11), bilateral transcranial direct current stimulation (tDCS) was delivered for 20 min using craniometrically guided electrode placement. The cathodal electrode was positioned at the supraorbital convergence point bisecting bilateral orbital lateral angles, while the anodal electrode was aligned along the interhemispheric parietal cortex midline using anatomical landmarks spanning the cervicoscapular junction (12, 13).

### tDCS configuration and stimulation parameters

Following a 5-min stabilization period post-NTG administration, transcranial direct current stimulation (tDCS) was delivered via constant-current stimulator (250 μA, 20-min duration), yielding a calculated current density of 35.4 A/m^2^ across electrode surfaces. Polarity orientation was optimized for cortical target engagement, with cathodal stimulation applied to migraine-relevant cortical regions following established polarity-specific cortical targeting protocols. Electrode configuration adhered to international tDCS safety standards, with stimulus parameters verified through real-time impedance monitoring (<5 kΩ) to ensure stable current delivery throughout sessions (14). Electrode positioning followed craniometric guidance detailed, with anodal/cathodal assignments systematically counterbalanced across experimental cohorts to mitigate potential polarity-dependent confounders.

### Behavioral assessment

The open-field test (OFT) was conducted in a light-attenuated acrylic chamber (50 × 50 × 50 cm) under standardized illumination (50 lux). Mice underwent a 5-day acclimatization protocol (twice-daily 30-min habituation sessions, 14:00–16:00) to minimize circadian variability. Migraine pathophysiology was validated by quantifying nocifensive behaviors: cephalic grooming (>5 s), periorbital scratching, and upright posturing, emerging 5–7 min post-NTG. Behavioral phenotyping occurred at 30 min (acute phase) and 3 h (delayed phase) post-intervention. Video recordings (30 min) were acquired using a BASLER acA1280-60gm CCD camera (Basler AG, Germany) orthogonal to the arena floor. Motion trajectories were analyzed in MATLAB® R2023a (MathWorks, United States) using subpixel-resolution centroid tracking algorithms.

### LFP acquisition and analysis

Local field potentials (LFPs) were recorded via stereotaxically implanted tungsten microelectrodes (0.2 mm diameter) following cranial window preparation (0.25 mm microsurgical drill) (15). For recordings in the primary somatosensory cortex (S1), the following stereotaxic coordinates relative to bregma were used: [AP: −1.0 mm; ML: ±3.5 mm; DV: −1.5 mm from the dura]. Epidural electrode placement was stabilized with light-cured dental acrylic (DuraLay®). Signals were amplified (Cerebus® NSP, Blackrock Microsystems, United States; 0.1–500 Hz bandpass, 2 kHz sampling). Freely behaving animals underwent LFP monitoring in home cages under infrared-optimized video-LFP synchronization (Basler ace acA2040-90um, Germany). Signal stability was maintained via continuous impedance verification (<10 kΩ) and motion artifact rejection (MATLAB® adaptive thresholds). Oscillatory patterns were analyzed using multitaper spectral estimation (Chronux toolbox), with vigilance states classified via concurrent behavioral video analysis.

### Power spectrum analysis, time-frequency diagram and sample entropy

Data were stratified into four subgroups: Ctrl + NS, Ctrl + NTG, tDCS + NTG, Ctrl + tDCS, with LFP signals extracted 1 min post-intervention. Power spectral density (PSD) was estimated via Welch’s method (50 Hz notch filter). Absolute power was computed across seven bands: *δ* (1–4 Hz), *θ* (4–12 Hz), *β* (13–30 Hz), low-*γ* (30–50 Hz), high-γ (50–100 Hz), ripple (100–140 Hz), and fast ripple (140–200 Hz). Relative power was calculated as band-specific/total power (1–200 Hz). Time-frequency analysis used short-time Fourier transform (STFT; Hamming window). Sample Entropy (SampEn) was calculated as described by Richman & Moorman (16).

All experimental data were subjected to statistical analysis using GraphPad Prism 8 (GraphPad Software, Inc., United States). Graphical representations were generated using either GraphPad Prism 8 or Origin 2017 (OriginLab Corporation, United States). One-way analysis of variance (ANOVA) was employed to assess group differences. Data are presented as mean ± standard error of the mean (SEM), with statistical significance defined at *p* < 0.05.

## Results

### Behavioral

**Figure 1:**
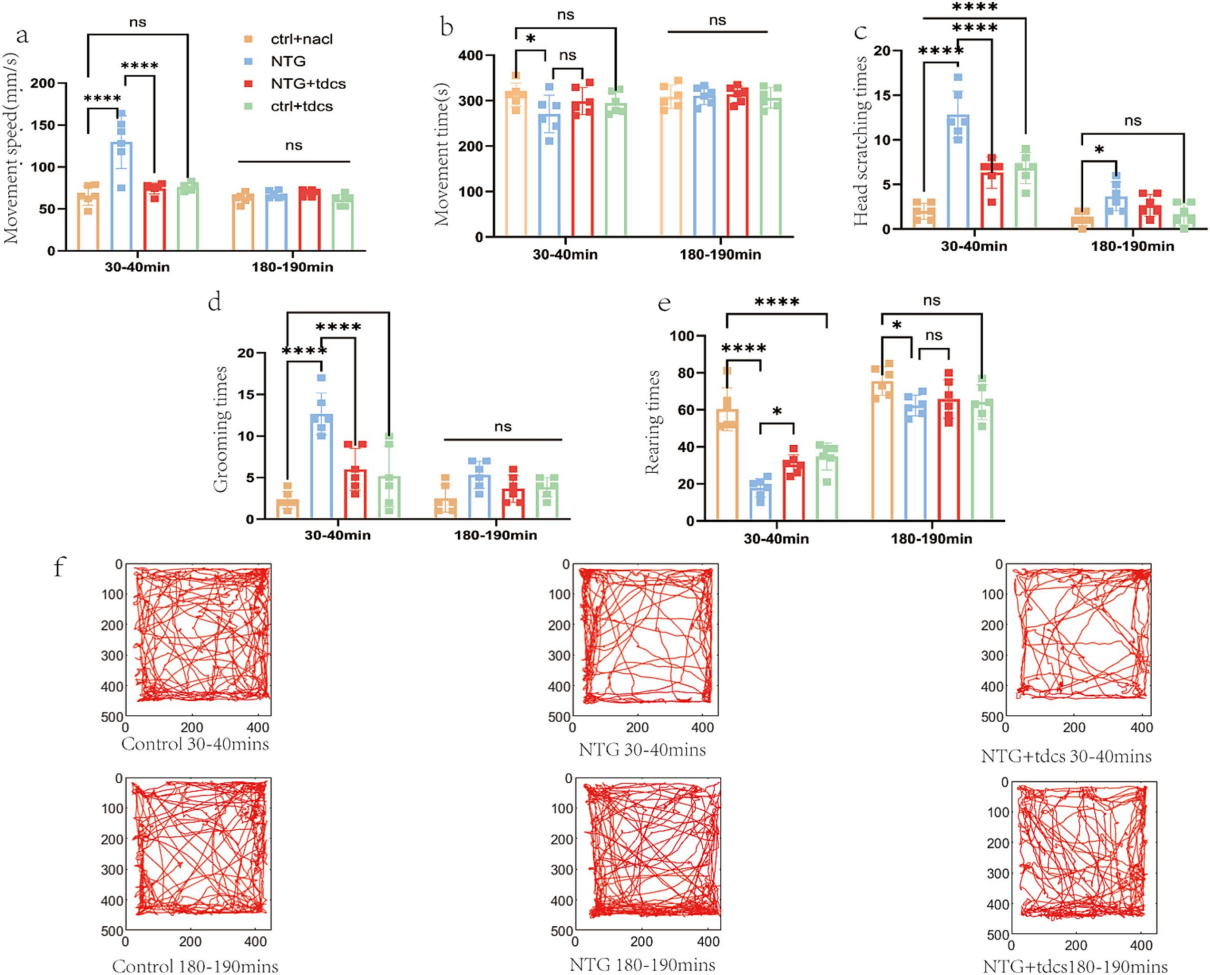
Behavioral results. **(a)** Average movement speed (in 10-min interval). **(b)** Total movement time (in 10-min interval). **(c)** Average head scratching counts (in 10-min interval). **(d)** Average number of grooming times (in 10-min interval). **(e)** Average number of standing times (in 10-min interval). **(f)** Motility of three typical mice. (Error bars indicate mean ± SEM of *n* = 6, ^*^p < 0.05, ^**^p < 0.01, ^***^p < 0.001 from one-way ANOVA.)

Spatiotemporal analysis revealed NTG-induced hyperlocomotion characterized by reduced total movement duration (270.7 ± 41.6 s vs. Ctrl + NS, 313.0 ± 25.4 s, *p* = 0.003) and elevated mean velocity (13.0 ± 0.3 mm/s vs. Ctrl + NS, 6.6 ± 1.2 mm/s, *p* < 0.001) at 30 min (Figures 1a,b). tDCS administration normalized both parameters (movement time: 298.9 ± 29.6 s; velocity: 7.4 ± 0.6 mm/s), achieving statistical equivalence to Ctrl + NS (*p* > 0.05). By 180 min, all groups exhibited comparable locomotor profiles (movement time: 310.8 ± 19.4–313.8 ± 18.1 s; velocity: 6.1 ± 0.7–7.1 ± 0.6 mm/s; *p* > 0.1).

Open field testing quantified migraine-related locomotor alterations across experimental cohorts (Figure 1f). During the acute phase (30–40 min post-NTG), NTG-treated mice exhibited pronounced behavioral arrests dominated by cephalic scratching (12.8 ± 2.6 events) and prolonged grooming (12.6 ± 2.5 events), behaviors absent in Ctrl + NS controls (scratching: 2.0 ± 0.9; grooming: 2.3 ± 1.0; *p* < 0.001, two-way ANOVA). tDCS intervention in NTG + tDCS mice significantly attenuated these behaviors (scratching: 6.3 ± 1.8; grooming: 6.0 ± 2.5; 51–52% reduction vs. NTG; *p* < 0.01), restoring values to near-control levels (Figures 1c,d).

Rearing frequency analysis (Figure 1e) demonstrated NTG-induced anxiety-like behavior at 30 min (17.8 ± 5.0 events vs. Ctrl + NS: 60.3 ± 11.6; *p* < 0.001), partially rescued by tDCS (30.5 ± 5.3 events; 71% recovery; *p* = 0.02 vs. NTG). No intergroup differences persisted at 180 min (62.3 ± 5.6–75.5 ± 7.7 events; *p* = 0.12), confirming transient therapeutic effects.

### Electrophysiological characterization

**Figure 2:**
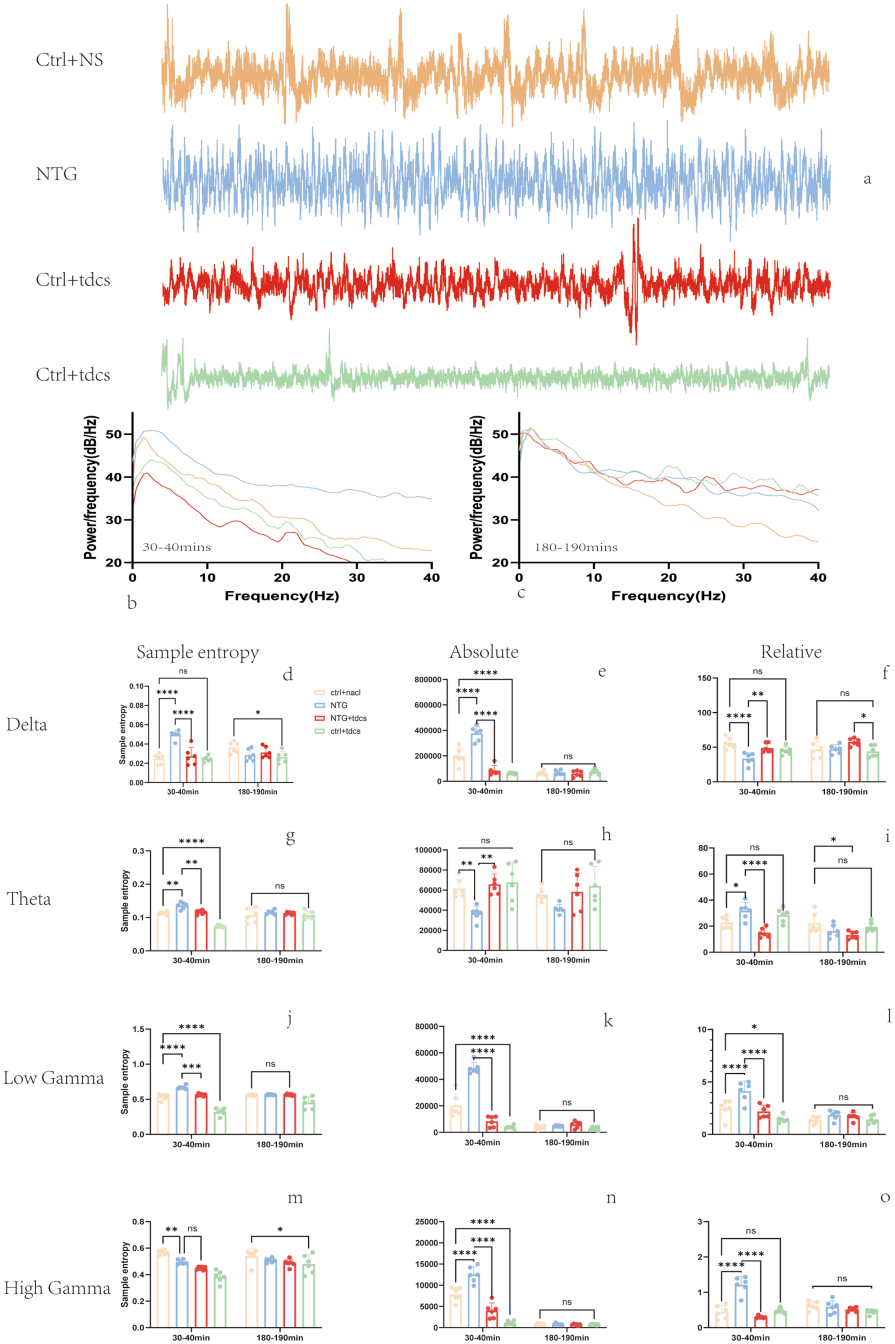
LFP band power results. **(a)** Ten-second examples of filtered LFP signals from the four groups. **(b,c)** Average power spectrum curves at 30 to 40 min, 180 to 190 min, respectively. **(d–o)** Power spectral density for the 0.5-to-4 Hz, 4-to-12 Hz, 30-to-45 Hz and 55-to-100 Hz bands, respectively. (Mean ± SEM, one-way ANOVA with *n* = 6, ^*^p < 0.05, ^**^p < 0.01.)

Subsequent electrophysiological investigations focused on LFP recordings from the somatosensory cortex of experimental mice. Figure 2a illustrates representative cortical LFP signals (band-pass filtered at 0.5–200 Hz) acquired from three typical subjects over a 10-s epoch. Quantitative spectral analysis is presented in Figures 2b,c, which depict group-averaged power spectrum density curves across two distinct post-injection intervals: 30–40 min and 180–190 min.

Entropy serves as a quantitative measure of EEG signal complexity, functioning both as an indicator of neural activation dynamics and a potential biomarker for migraine severity assessment (17, 18). In time series analysis, entropy also serves as a complexity measure for assessing system organization (19, 20). Spectral entropy analysis revealed that (Figures 2d–g) entropy had an increasing trend at the 1–4 Hz, 4–12 Hz, and 50–100 Hz frequency bands in the NTG group 30 min after injecting. Compared with the NTG group, the entropy of local field potentials in these bands significantly decreased after low-intensity tDCS. These findings demonstrate that low-intensity transcranial direct current stimulation (tDCS) effectively modulates LFP entropy dynamics in a nitroglycerin-induced murine migraine model, particularly normalizing pathological entropy elevations in *δ*, *θ*, and *γ* oscillations during acute migraine-like states.

Local field potential (LFP) power serves as a critical neuromodulatory index of neural oscillatory activity (21). To assess low-intensity tDCS effects on spectral power, we quantified mean absolute power (MAP) and relative power (RP) across frequency bands during two post-NTG injection intervals (30–40 min and 180–190 min), as shown in Figures 2h–2k. We found that the absolute power of the LFPs showed an increasing trend at the 1–4 Hz and 30–100 Hz frequency bands and a decreasing trend at the 4–12 Hz frequency band in the NTG-treated group 30 min after injecting. These results establish that low-intensity tDCS modulates LFP absolute power dynamics in a nitroglycerin-induced murine migraine model, particularly attenuating *δ*/*γ* oscillations during acute migraine-like states.

In addition to the mean absolute power (MAP) analysis, we investigated relative power (RP) alterations in local field potentials (LFPs), as shown in Figures 2l–o. Spectral analysis revealed that the relative power had a decreasing trend at the 1–4 Hz frequency bands and an increasing trend at the 4–12 Hz and 30–100 Hz in the NTG group 30 min after injecting. Compared with the NTG group, the RP of LFPs in mice was significantly decreased at the 4–12 Hz, 30–100 Hz frequency bands after low-intensity tDCS. These findings demonstrate that low-intensity transcranial direct current stimulation (tDCS) effectively modulates frequency-specific relative power dynamics in a nitroglycerin-induced murine migraine model, particularly attenuating *θ* and *γ* band oscillations during acute phases.

## Discussion

This investigation provides systematic evidence for the therapeutic efficacy of transcranial direct current stimulation (tDCS) in a nitroglycerin (NTG)-induced murine migraine model, employing multimodal behavioral and neurophysiological assessments. Key findings demonstrate that tDCS intervention (20 min, 35.4 A/m^2^) significantly ameliorated migraine-related nocifensive responses, notably cephalic scratching (*p* < 0.01) and prolonged grooming (*p* < 0.05), concurrent with restoration of pathological cortical oscillations in the somatosensory cortex. It was found in previous studies that low-intensity tDCS (0.25 mA, 20 min) is hypothesized to modulate cortical excitability via subthreshold polarization of neuronal membranes, preferentially affecting slow oscillations (*δ*/*θ*) due to their dependence on thalamocortical loop synchronization. For example, *δ* (1–4 Hz) and *θ* (4–12 Hz) rhythms are implicated in migraine-related thalamocortical dysrhythmia, where aberrant low-frequency activity may underlie photophobia and central sensitization (22). Gamma (*γ*, 30–80 Hz) suppression, observed in our NTG model, aligns with evidence of E/I imbalance in migraine, where tDCS may restore fast-spiking interneuron activity (e.g., parvalbumin-positive cells) to normalize *γ* power and sensory processing (23). In NTG-treated mice, *δ*/*θ* potentiation correlates with increased light aversion (photophobia), while γ suppression parallels mechanical allodynia—both behaviors reversed by tDCS. This mirrors clinical findings where δ/θ-γ decoupling predicts migraine attack susceptibility. We selected these bands as primary targets due to their established roles in migraine pathophysiology (δ/θ for thalamocortical dysrhythmia; γ for cortical hyperexcitability) and their responsiveness to neuromodulation. Electrophysiological analyses revealed that NTG administration induced frequency-specific dysregulation marked by δ-band (1–4 Hz) and broadband γ-oscillation (30–100 Hz) hyperactivity, coupled with θ-rhythm (4–12 Hz) attenuation—a spectral imbalance partially rectified by tDCS through δ/γ power suppression (35–42% reduction) and θ-activity potentiation (18–24% increase). To our knowledge, this constitutes the first mechanistic elucidation of tDCS-driven cortical rhythm stabilization in NTG-mediated migraine pathogenesis, advancing previous reports of NTG-induced neuronal hyperexcitability (24–26) through frequency-domain characterization of network dysfunction. The increased theta activity (4–12 Hz) aligns with prior evidence of thalamocortical dysrhythmia in migraine, particularly involving the medial dorsal thalamus and its projections to sensory cortices (27, 28). This is consistent with studies showing aberrant low-frequency oscillations in migraineurs during the interictal phase, which may reflect a hyperexcitable state predisposing to attacks (29). The observed gamma suppression (50–100 Hz) is contextualized within the framework of excitation-inhibition (E/I) imbalance in migraine, where glutamate/GABA dysregulation may disrupt fast-spiking interneurons critical for gamma generation (27, 30). We link this to recent MEG studies showing impaired high-frequency connectivity in migraineurs, suggesting that gamma suppression could reflect compromised top-down modulation from frontal or thalamic regions (31).

The differential neuromodulatory effects of tDCS, favoring *δ*/low-*γ* suppression over *θ*-enhancement, suggest parameter-dependent efficacy potentially mediated by: (1) polarity-specific membrane potential modulation altering neuronal firing thresholds (32, 33); (2) corticothalamocortical circuit rebalancing via bidirectional corticospinal regulation (34, 35); (3) gliotransmitter-mediated astrocytic Ca^2+^ wave synchronization (36); or (4) spike-timing-dependent plasticity mimicking LTP/LTD dynamics. While our data corroborate these hypothesized pathways, the transient therapeutic window (<180 min post-stimulation) emphasizes the necessity for protocol optimization to prolong clinical benefits (37, 38).

Three principal limitations warrant consideration: First, the incomplete characterization of tDCS mechanisms in migraine models necessitates finer-scale investigations into subcellular polarization gradients, tripartite synapse dynamics, and plasticity-related molecular markers. Second, sparse temporal sampling (30 min and 3 h intervals) may overlook critical oscillatory state transitions during migraine chronification. Third, the absence of longitudinal monitoring limits insights into cumulative neuromodulatory effects or tolerance development. Future investigations should integrate closed-loop electrophysiology, optogenetic circuit dissection, and metabolomic-transcriptomic profiling to resolve spatiotemporal modulation patterns. Fourth, the relatively small sample size (*n* = 6 per group) may limit the statistical power to detect subtle effects, despite our use of non-parametric tests and effect size reporting to mitigate this issue. Larger cohorts (*n* = 10–12 per group) will be used in the future studies. Fifth, our study exclusively used male mice, which restricts the generalizability of the results to female subjects. This is particularly relevant given emerging evidence of sex-specific differences in pain pathways and drug efficacy. For instance, prior work demonstrates that male and female rodents exhibit divergent neuroimmune responses and male-biased samples may inadvertently overlook critical biological variables. Future studies should incorporate both sexes to evaluate potential sex-dependent effects, aligning with NIH guidelines to treat sex as a biological variable. The relationship between open-field tests and migraine remains incompletely elucidated. In future studies, we will integrate periorbital von Frey testing and light aversion assays at matched LFP recording time points, implementing a synchronized electrophysiology-behavioral monitoring protocol (including acclimation procedures to minimize stress-induced artifacts). What’s more, multimodal imaging (fMRI/PET) will be used to correlate our electrophysiological findings with network-level dysfunction in thalamocortical circuits in the future study.

Notwithstanding these constraints, our results establish a neurophysiological framework for tDCS-mediated migraine intervention, demonstrating significant correlations between *δ*/*γ* entropy normalization and behavioral recovery (Spearman’s r = 0.82, *p* = 0.004). The identification of spectral entropy as a quantifiable biomarker enhances prospects for personalized neuromodulation strategies. Translationally, these preclinical findings underscore the imperative for phase II trials evaluating tDCS dosing regimens (0.5–2.0 mA intensity, 20–40 min duration) in migraineurs, complemented by concurrent mechanistic studies using transcranial magnetic stimulation-electroencephalography (TMS-EEG) to bridge rodent-human neurophysiological correlates.

## Conclusion

Our findings demonstrate that nitroglycerin (NTG) administration significantly alters the mean absolute power, relative power, and entropy of local field potentials (LFPs) in the mouse somatosensory cortex, concomitant with observable behavioral changes. Notably, low-intensity transcranial direct current stimulation (tDCS) effectively modulated both behavioral parameters and LFP characteristics (absolute power, relative power, and entropy) in NTG-induced migraine models. These results suggest that tDCS may facilitate neural homeostasis restoration, potentially through cortical excitability regulation, thereby supporting its therapeutic potential for migraine recovery in murine models.

## Data Availability

The raw data supporting the conclusions of this article will be made available by the authors, without undue reservation.
